# Identifying Neurobiological Underpinnings of Two Suicidal Subtypes

**DOI:** 10.20900/jpbs.20210016

**Published:** 2021-08-31

**Authors:** Barbara Stanley, Liat Itzhaky, Maria A. Oquendo

**Affiliations:** 1 Division of Molecular Imaging and Neuropathology, New York State Psychiatric Institute, Unit 42, 1051 Riverside Drive, New York, NY 10032, USA; 2 Columbia University College of Physicians and Surgeons, New York, NY 10032, USA; 3 Perelman School of Medicine, University of Pennsylvania, 3535 Market Street, Suite 200, Philadelphia, PA 19104, USA

**Keywords:** suicide, suicidal ideation, phenotypes

## Abstract

Despite substantial suicide prevention efforts, US suicide rates continue to climb, currently reaching about 14 per 100,000 individuals. Suicidal behavior has been linked to neurobiological, neurocognitive and behavioral factors; however, integrative, multi-modal studies are rare. Furthermore, prospective studies, crucial to understanding future risk factors, have focused on a single predictor and a single outcome, implying that suicidal behavior is homogeneous. But recent research shows suicidal behavior is complex and heterogeneous, with the possible existence of subtypes. The present report describes a project testing a model that posits two putative subtypes, using a prospective, multi-model design. The subtypes differ in regard to the patterns of suicidal ideation and underlying mechanisms. One hundred subjects diagnosed with a Major Depressive episode, half of whom have attempted suicide in the past, are enrolled and followed for two years, notably the highest risk period for suicidal behavior. Baseline assessments include a clinical assessment, neurocognitive and behavioral tasks, Ecological Momentary Assessments (EMA), PET imaging, and a cognitive emotion regulation task in the MRI scanner. The follow-up assessment includes a clinical assessment and EMA. The study findings have the potential to pave the way for a clearer understanding of suicidal ideation and behaviors and to improve our ability to treat those at risk for suicide by developing tailored approaches that will allow for more accurate pharmacological and psychosocial interventions.

## INTRODUCTION

Suicide is one of the leading causes of death in the US. Despite extensive research efforts to understand and prevent suicide, there was a 33% increase in suicide deaths from 1999 to 2017 [1]. One reason for the failure in reducing suicide rates could be the underlying assumption in most studies and clinical approaches that suicidal behavior is a homogeneous outcome. However, suicidal behavior is complex and heterogeneous. It can be impulsive [2,3] or methodically planned [4]; violent or not [5]; reactive to stress or occur with no obvious stressors [6]. Therefore, it may be more fruitful to define different subtypes of suicidal behavior, allowing for further understanding of the specific underlying mechanisms. Such a person-centered approach will allow for more accurate suicide prevention research and treatment.

**Figure 1.**
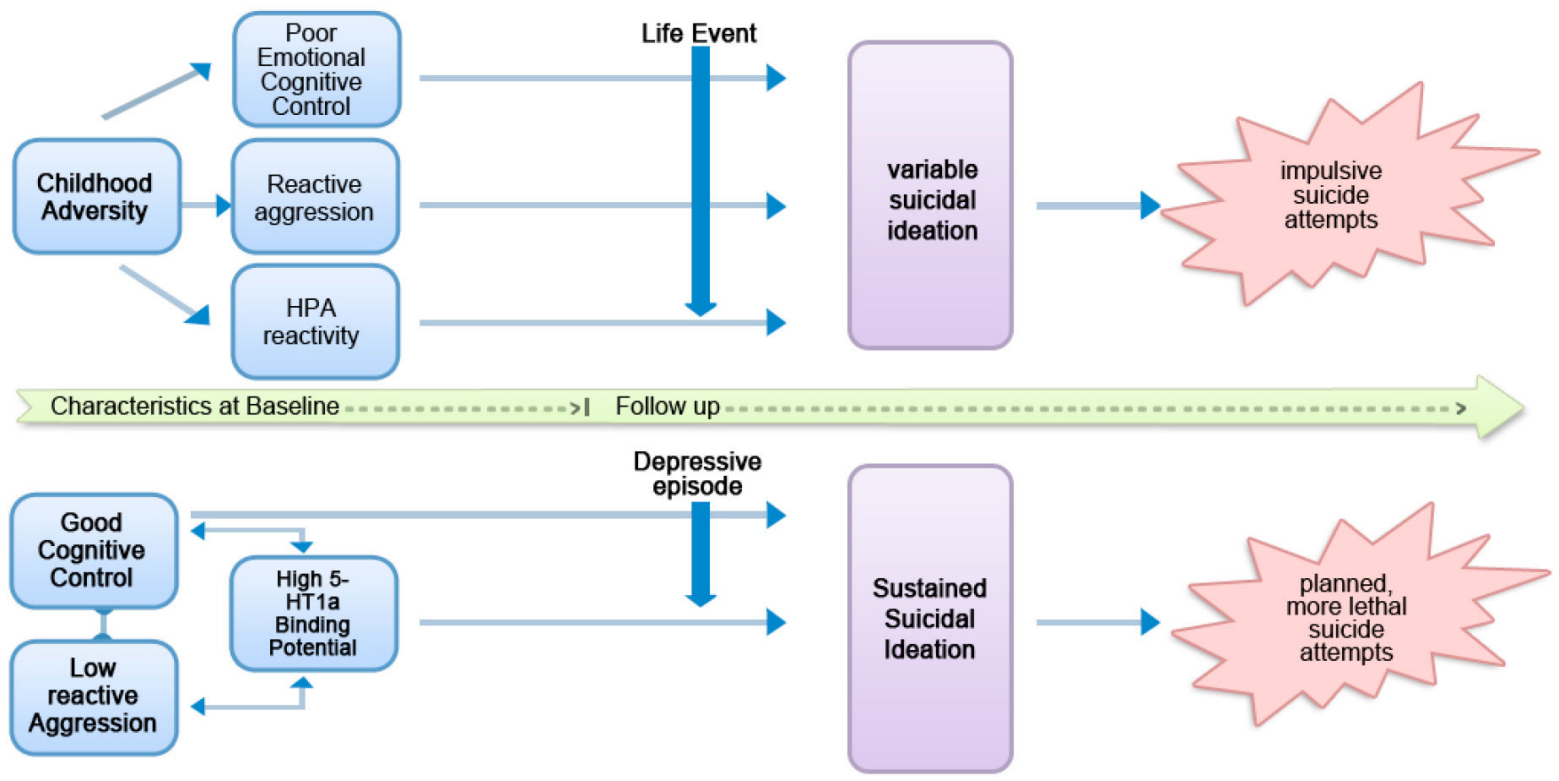
Explanatory model for two subtypes of suicidal behavior [9].

Studies have previously delineated an impulsive [2,3,7] and a planned [8] suicidal behavior subtypes. To our knowledge there have been no prospective studies examining predictors of suicidal ideation subtypes. In the current study, we are testing an integrative, multi-level model (Figure 1), which posits two putative subtypes of suicidal behavior associated with different patterns of suicidal ideation. On the one hand, there is a suicidal ideation variable pattern that fluctuates greatly over short periods and leads to impulsive suicidal behavior, typically in response to environmental stressors. This variable suicidal ideation pattern is speculated to occur in those with a trauma history, high reactive aggression, pronounced cortisol response to stress and difficulty engaging prefrontal regions in affect regulation. In contrast, when suicidal ideation is elevated but with little fluctuation, we postulate that it is linked to blunted serotonergic function, greater cognitive control and more planned, lethal suicidal behavior.

## THE PROJECT'S SPECIFIC AIMS AND HYPOTHESES

### Aim 1.

To identify neurobiological, neurocognitive and behavioral features linked to suicidal ideation variability during Ecological Momentary Assessment (EMA) follow-up.

H1.1. Higher reactive aggression, greater cortisol response to a psychosocial stress, poorer cortical control (orbital prefrontal cortex and precuneus) during emotion regulation tasks and history of childhood trauma will predict greater suicidal ideation variability at follow-up.

H1.2. Childhood trauma's effect on suicidal ideation variability will be mediated through a combination of baseline reactive aggression, greater cortisol response to stress, and poor emotional cognitive control.

H1.3. Higher reactive aggression, greater cortisol response to a psychosocial stress, poorer cortical control (orbital prefrontal cortex and precuneus) during emotion regulation fMRI tasks and history of childhood trauma will predict greater suicidal ideation increases after life events, both measured by EMA during follow-up.

EXP. H.1.1. Explore whether suicidal ideation variability is consistent across EMA epochs.

### Aim 2.

To identify neurobiological features associated with suicidal ideation that is not variable (negatively associated with suicidal ideation variability) as measured by both EMA and clinical assessments during follow-up.

H2.1. Higher baseline 5HT1A BPF in Dorsal Raphe Nucleus as measured by PET, indicating low serotonergic function, will be negatively associated with suicidal ideation variability, during follow-up.

EXP. H.2.1. Explore whether baseline Dorsal Raphe Nucleus 5HT1A BPF predicts (A) degree of planning for SB during follow-up; (B) future high lethality SB. A and B will be measured with clinical scales.

EXP. H.2.2. Explore whether baseline cognitive control [Stroop, Continuous Performance Tasks] is (A) correlated with DRN 5HT1A BPF; (B) predictive of high mean suicidal ideation, but low suicidal ideation variability at baseline and follow up EMA and (C) predictive of chronic suicidal ideation measured by clinical scales.

### Impact.

In large general population samples, most suicide attempters report suicidal ideation (NESARC [94.2%]; NLAES [86.8%]) [10], yet >50% of suicides had no prior SB [11]. Thus, prior suicidal behavior is limited as a predictor of future suicidal behavior, especially suicide death; better understanding of suicidal ideation is crucial to improving prediction and understanding of suicidal behavior. Critically, if variable suicidal ideation is a harbinger of impulsive suicide attempts, then delineation of associated distinct biological and clinical features, may help delineate different suicidal behavior phenotypes and risk patterns. Ultimately, these data may aid in prospectively identifying those at risk for different expressions/phenotypes of suicidal behavior. Moreover, future studies could examine the merits of pharmacologic or psychological interventions targeting affect regulation for variable suicidal ideation, versus antidepressants or cognitive treatments for sustained suicidal ideation.

### Significance.

Despite unwavering efforts to prevent suicide, US rates are climbing and are currently about 14 per 100,000. Similar trends in suicide attempt rates are also observed [1]. Remarkably, 90% of suicides have a mental disorder [12] and those with depression are both 70% more likely to die by suicide than the general population [13,14] and more likely to attempt suicide than those with other psychiatric disorders [14,15]. Finally, psychological autopsy studies report that 60% of suicides were in a Major Depressive Episode [16,17]. Thus, studying risk factors for suicidal behavior in depressed individuals is essential.

### Innovation.

Research suggests suicidal behavior is not just a symptom of a psychiatric diagnosis [18], rather, it has its own neurobiological and neurocognitive underpinnings. While studies have found aggression, serotonin dysfunction, hypothalamic-pituitary-adrenal axis dysregulation and childhood trauma to be related to suicidal behavior [19], effects are typically modest. Most models posit a single path to suicidal behavior that all suicidal individuals follow. We suggest that there are at least two different subtypes of suicidal behavior, related to difference patterns of suicidal ideation. By conflating the two subtypes, predictors’ effects may have been diluted or worse, yielded contradictory outcomes.

Our project is innovative in several ways:

1. We posit two paths to suicidal behaviors through variable suicidal ideation and sustained suicidal ideation. Importantly, if confirmed, this may affect a paradigm shift leading to delineation of multiple subtypes of suicidal behavior, each with a unique set of neurobiological, neurocognitive and environmental attributes.
2. This is one of the first multi-modal (fMRI, PET, biobehavioral), prospective study of suicidal ideation subtypes using state-of-the-art neurobiological, neurocognitive and behavioral measures allowing quantification of their relationships.
3. This is also one of the first studies to use an emotion regulation cognitive control fMRI paradigm to predict suicidal ideation and suicidal behavior subtypes.
4. The use of EMA to evaluate moment to moment fluctuations in affect, suicidal ideation and stressors is innovative. EMA provides a rich source of data that circumvents recall bias and enables close dissection of the interplay of stressors, affect, suicidal ideation and behaviors in ways that standard assessments cannot.

## METHODS

### Participants.

Participants (*n* = 100) are physically healthy inpatients or outpatients of New York State Psychiatric Institute (NYSPI), New York Presbyterian Hospital or Columbia University Irving Medical Center (CUIMC). Inclusion criteria are major depressive episode; for half of participants suicide attempt (defined as having a history of a self-destructive act with intent to die and medical lethality of >2); age range 18–60 years; right-handed; normal cognitive function; off psychotropic medication or clinically significant symptoms present despite adequate dose and duration of medication treatment. Exclusion criteria are significant active physical illness; movement disorders except familial tremor; history of closed head trauma with loss of consciousness; history of cerebrovascular disease (stroke, TIA); abnormal MRI (except changes accounted for by technical factors); lack of capacity to consent; pregnancy; history of alcohol or substance abuse or dependence in the last 6 months; claustrophobia; metal in body or prior history working with metal fragments (e.g., as a machinist); any other contraindications for MRI examination (e.g., metallic implants such as pacemakers, surgical aneurysm clips, or known metal fragments embedded in the body).

### IRB Review.

The study was approved by the New York State Psychiatric Institute Institutional Review Board (Protocol # 6776; approved 6/25/2013). Informed consent is obtained from all participants. Because this study is conducted in a high risk group, we provide additional participant emergency contact and care. While we use EMA in this project, we inform our participants that we do not actively monitor their assessments and let them know that should they find themselves in crisis or with increasing suicidal ideation, they should contact our 24/7 on call psychiatrist. This procedure helps to avoid unnecessary emergency room visits or calls to 911. At the same time, it helps to maintain the integrity and validity of the data by not contacting participants each time their suicidal ideation increased and resulting in reactivity in the findings. We believe that there is sound clinical and scientific grounds for this approach given the patterns of suicidal ideation, particularly those that are highly variable. Individuals with highly variable ideation spike up and remit very quickly and it is atypical for them to reach out or feel the need for clinical care during a spike. This procedure works well in our EMA studies because participants are assigned a psychiatrist who sees them over the course of the study and is available to them on an emergency basis in addition to the on call psychiatrist.

### Procedure.

Baseline clinical, EMA and behavioral assessments are administered at enrollment when participants are depressed. Participants are followed up at 3, 6, 12, 18 and 24 months using EMA and at 3, 12 and 24 months using standard clinical assessments.

Assessments are conducted blinded and reviewed within a week of enrollment. Consensus diagnoses are generated using all information available at weekly consensus conferences, based on structured instruments with special attention to comorbidities. Upon study admission, tracking data are recorded including the names, phone numbers, and addresses of at least three family members or friends to contact. If we lose contact, we use commercial databases that normally assist in debt collections and provide current addresses. Participants are compensated for their time and expenses.

#### Baseline Assessments

##### Clinical measures

###### Suicidal Behavior:

Assessment of suicidal behavior is based on C-CASA algorithm [20] using the Columbia Suicide Severity Rating Scale (C-SSRS) [21]. The C-SSRS is a semi-structured interview that elicits history of suicidal ideation, plans, attempts and circumstances surrounding suicidal behaviors. As data supports a continuum of suicidal behavior predicting risk for a future suicide attempt [22,23] we included suicide-related behaviors (e.g., aborted attempt, defined as a behavior in which an individual begins a suicide attempt but decides not to proceed).

The Scale for Suicidal Ideation (SSI) [24] is used to assess active and passive suicidal ideation and planning. A comprehensive family history of suicidal behavior is assessed using the Family Interview for Genetic Studies (FIGS) [25]. Suicide attempters only are also completing the Suicide Intent Scale (SIS) [26] and Lethality Rating Scale (LRS) [27].

###### Psychiatric Diagnoses:

Axis I and II DSM-IV diagnoses are evaluated using the Structured Clinical Interview for DSM-IV (SCID I and II, Patient versions) [28].

###### Childhood and Lifetime Adversity:

Childhood trauma is evaluated using the Childhood Trauma Questionnaire (CTQ) [29], a self-report questionnaire assessing childhood physical and sexual abuse and neglect, as well as our Demographic form that documents physical or sexual abuse, death of a parent, separation from parents, adoption and orphanage data < age 15 years. Recent life events are assessed with the Recent Life Changes Questionnaire [30]. Family psychiatric history is obtained via the FIGS [25].

###### Depression:

Depression severity is assessed using clinician rated and self-report measures: Hamilton Rating Scale for Depression (HDRS) [31] and Beck Depression Inventory (BDI) [32].

###### Demographic Data:

Demographic Form collects demographic information, history of psychiatric treatment including drug trials, hospitalizations, medical history, head injury, and menstrual history, adding to the characterization of the sample.

###### Neurocognitive Testing:

To assess cognitive/attentional control, we use the Continuous Performance Test (CPT) [33] and the Computerized Stroop Task (CST) [34].

###### Behavioral Paradigms:

To evaluate aggressive behavior, we use the Point Subtraction Aggression Paradigm (PSAP), which is a standardized aggression provocation behavioral task. To assess cortisol response to social stressors we use the Trier Social Stress Test (TSST) [35].

###### Ecological Momentary Assessment (EMA):

To monitor fluctuations in suicidal ideation, suicidal behavior, life events, positive/negative affects, stressors and emotions, subjects are given an electronic device (iPOD) for assessments for 7 days/6× per day/<6 min/time at enrollment. If the subject records the presence of suicidal ideation, an open-ended question allowing the subject to record descriptions of their experience is presented. Subjects are all reminded about emergency procedures to contact staff if they are concerned about suicidal ideation or risk.

##### fMRI methods

###### Emotion Regulation Task.

Participants perform two fMRI tasks: (1) recall of eight recent upsetting autobiographical memories described in detail in advance with an interviewer and then cued in the scanner; (2) viewing emotional pictures from the International Affective Pictures System (IAPS) [36]. In the IAPS task, selected pictures depict people (usually two persons interacting) in emotionally evocative situations. These two tasks increase both external (personal memories task) and internal validity (standardized IAPS pictures).

On both tasks, there are two types of trials. On trials assessing baseline affective responses, subjects immerse themselves in the image or memory. On regulation trials, subjects increase their sense of objective distance by viewing pictured events or memory from a detached, third-person perspective. Prior to scanning, subjects receive training and practice in the distancing technique, which includes describing the cognitive processes they employ with successive shaping of their cognitions by the trainer until the technique is mastered. During scanning, the IAPS task includes 5 blocks of 18 trials, each consisting of a visual instructional cue (2 s) to “distance” or “immerse” from a subsequently presented photos (8 s). The recall task includes 4 blocks of 4 trials, each consisting of a recall cue (10 s) followed by an instructed elaboration period (20 s) where each memory is re-experienced from the immersed or distanced perspective, as instructed. Ratings of negative affect (3 s) are collected at the end of each trial for both tasks and ratings of vividness are collected at the end of each trial for the memory paradigm (3 s). All trial events and inter-trial intervals are jittered (1–7 s intervals).

###### Image Acquisition.

Images are acquired using a GE MR750 3.0 Tesla magnet, equipped with a 32-channel system and an EXCITE headcoil, which support parallel imaging.

###### Image Analysis.

Preprocessing is conducted using SPM8 (Welcome Department of Cognitive Neurology, London) and first and second-level analyses are carried out using NeuroElf (https://neuroelf.net/) under Dr. Ochsner’s supervision using methods well-established within his laboratory and described in more detail elsewhere. An event-related analysis models the hemodynamic response for critical task-related regressors (e.g., for both tasks instruction and picture viewing/memory recall separately for immerse and distance trials, plus affect rating). At each scan, participants are assessed for smoking on the day of testing, hematocrit, estradiol, heart and breath frequency and intake of any vasoactive substances (e.g., caffeine, benzodiazepines).

##### PET acquisition

Briefly, preparation of [^11^C]CUMI-101, image acquisition and analysis are conducted as previously described [37]. A radial artery catheter will be placed for blood sampling to obtain a metabolite-corrected arterial input function. Free-fraction of all radioligands in plasma will be measured. The cerebellum will serve as the reference region. Regional distribution volumes (V_T_) will be derived from kinetic analysis using a metabolite-corrected arterial input function. Several compartmental models will be considered. BPF will be calculated as (V_T_ -V_ND_)/*f*P, where V_ND_ = distribution volume of the reference region and *f*P = plasma free-fraction.

#### Follow Up Assessments

##### Assessments.

Follow-up clinical assessments are administered at 3, 12 and 24 months by phone by the clinician best known to the subject or, if preferred, in the clinic, and at 3, 6, 12, 18 and 24 months for EMA. The EMA assessment allows us to closely track patterns of suicidal ideation, depression, stressors and affects as well as suicidal behavior over this high-risk period.

##### Suicidal Ideation during Follow-up.

The principal outcome measure is derived from 5 discrete week-long EMA (3, 6, 12, 18, 24 months), using a 9-item suicidal ideation (5-point Likert) scale to calculate suicidal ideation variability. Secondary outcomes for exploratory hypotheses are based on SSI subscales such as the planning subscale, or items from the SSI [24]. Additional outcomes for exploratory hypotheses include: attempts since the last evaluation as recorded on the Columbia Suicide History Form [38]. The SIS [26] and the LRS [27] are completed for each attempt. The SSI [24] and the C-SSRS [21] are completed regardless of whether suicidal behavior occurred. SSI is rated for the prior two weeks and the two weeks with the worst suicidal ideation during an intervening MDE, if any.

##### Major Depression during Follow-up.

On follow-up, the number of episodes meeting syndromal diagnostic criteria for MDE and their duration since the previous visit are being assessed using the DSM-IV SCID and Psychiatric Follow-up Form. We assess the presence of an MDE on a month-to-month basis, marking it as present or absent. We have demonstrated the utility of this approach showing that the hazard of suicidal behavior increases thirteen-fold when there is an MDE in the follow-up period [39]. Dimensional measures of symptoms are assessed at 3, 12 and 24 months by HDRS [31] and BDI [32].

##### Life Events during Follow-up.

We generate a single score derived from the Recent Life Changes Scale [30]. Life events are also being measured by EMA at 5 discrete follow-up time-points.

## STATISTICAL ANALYSIS

The primary measure of suicidal ideation will be the sum of the suicidal ideation EMA items during 5 discrete week-long EMA periods at 3, 6, 12, 18, 24 months. Suicidal ideation variability will be measured during follow-up for each EMA epoch separately and defined as the standard deviation of the suicidal ideation values (SDSI*_ik_*) where i indicates subject and k indicates epochs.

Aim 1. H1.1 and 1.2. Associations between suicidal ideation variability measures (derived as above for periods with suicidal ideation >0 as measured by EMA), and baseline predictors: Reactive Aggression (RA), cortisol response to TSST (TSSTCR), cortical control (CONTR), childhood trauma (TRAU) will be tested using a single predictor as well as a multiple predictor mixed effect regression model with the subject-specific variation measures (repeated measures per subject) as outcome, with the baseline predictors as fixed predictors and subject-specific random intercepts, with an AR correlation structure: (1) SDSI*_ik_* = β _0_ + β_1_*RA*_i_* + β_2_*TSSTCR*_i_* + β_3_*CONTR*_i_* + β_4_*TRAU*_i_* +ε*_ik_*, for *i* = 1,..., *n*, *k* = 0,…, K*_i_* - 1 where K*_i_* is the number of EMA epochs for subject *i* when suicidal. We hypothesize that the first three predictors will have significant independent effects on suicidal ideation variability. However, while TRAU will be a significant predictor of suicidal ideation variability in a single predictor model, in the adjusted model we do not expect it to be significant. The mediation relationship will be tested using the joint model rather than single-mediator models, because we expect the mediators to be correlated with each other, even adjusting for childhood trauma, and thus we will adjust each variable’s effect on the outcome. We will estimate the indirect effect of TRAU on SI variability that is mediated through the other three predictors and will compare it to the complete effect, i.e., the effect when only TRAU is in the model. A 95% confidence interval (CI) for the total indirect effect will be calculated using the bootstrap. In secondary analyses, we will explore exposure-mediator and mediator-mediator interactions. For H1.3, the outcome of interest is change in suicidal ideation after a life event. We will fit 4 mixed effect regression models similar to the equation below, with baseline predictors interacting with the time-varying life event indicator variable: (2) SI*_ij_* - SI*_i_*_(_*_j_*_−1)_ = β_0_ + β_1_* Event*_ij_* + β_2_* RA*_i_* + β_3_* Event*_ij_* *RA*_i_* + ε*_ij_*, *i* = 1,…, *n*, *j* = 1,…,p*_i_*, where p*i* denotes the number of EMA observations from all EMA epochs for subject *i*. The coefficient of interest will be β_3_. Observations from all EMA epochs with suicidal ideation > 0 will be used. Change scores in suicidal ideation within 24 h of the event will be calculated for follow up time points. The model will include subject-specific random intercepts and coefficients for the event indicator variable. For EXP. H. 1.1, we will fit a mixed effect regression model similar to (1) above, without the baseline predictors but including a categorical predictor for timepoint, and we will test the coefficient of the (categorical) time variable.

Aim 2: For H2.1, log-transformed PET 5HT1A binding in the DRN will be entered as predictor into a mixed effect regression model with the longitudinally measured suicidal ideation variability as outcome variable and subject-specific random intercept. For testing all ROIs simultaneously, we will aggregate the variability measures into one value and use it as the predictor in a mixed effect model with (log) binding values as outcome, where ROIs are the within-subject effects. In EXP. H.2.1A, the outcome will be the score on the longitudinal planning subscale of the SSI measured at Follow-up visits, and the analysis will use mixed effect regression models similar to those described above. In EXP. H.2.1B, we are interested in the maximal lethality of future SB from the Beck Lethality Rating Scale during follow-up. We will use a weighted regression model with PET binding in the DRN, weighted by the standard error of the measurement as the response variable and the lethality score as the predictor. For EXP. H.2.2A, the association between baseline neurocognitive control measures (CPT and Stroop) and PET binding will be tested using weighted regression models as described in EXP. H.2.1B; then two mixed effect logistic regressions will be fit with neurocognitive control measures as the predictors and these outcome variables: (a) a longitudinal indicator variable denoting EMA epochs with high mean and low variability suicidal ideation; and (b) a longitudinal indicator variable of chronic ideation derived from item 6 of the SSI during follow-up.

## SUMMARY AND LIMITATIONS

Suicide rates are increasing despite tremendous efforts invested in the research of suicide phenomena and in developing prevention programs, therefore a paradigm shift may be helpful. As suicide is a heterogeneous phenomenon, the ongoing project we report on here aims to define subtypes of suicidal behavior, using a multi-model design of clinical and biobehavioral assessment measures, fMRI, PET as well as EMA. This methodologically rich approach of in-depth investigation will allow a better understanding of the diverse underlying mechanisms of suicidal behavior and consequently more personalized suicide prevention interventions.

One of the advantages of the current study also imposes certain limitations. The inclusion of only individuals with MDD in the sample may limit the generalizability of the study’s findings and may also limit our power to detect suicidal behavior subtypes (as the variance within individuals with MDD may be smaller than in the general population). Furthermore, because the study is confined to MDD, we will not know if the subtypes apply to other diagnostic groups in which suicidal behavior occurs at a high rate, e.g., bipolar disorder. We do, however, have data that support the existence of these subtypes in borderline personality disorder [40], another high risk group. However, we believe that the strength of this approach outweighs the limitations in regards to the important contribution to the field of suicidology.
